# A multi-centric dataset on patient-individual pathological lymph node involvement in head and neck squamous cell carcinoma

**DOI:** 10.1016/j.dib.2023.110020

**Published:** 2023-12-29

**Authors:** Roman Ludwig, Adrian Schubert, Dorothea Barbatei, Laurence Bauwens, Sandrine Werlen, Olgun Elicin, Matthias Dettmer, Philippe Zrounba, Panagiotis Balermpas, Bertrand Pouymayou, Vincent Grégoire, Roland Giger, Jan Unkelbach

**Affiliations:** a Department of Radiation Oncology, University Hospital Zurich, Rämistrasse 100, 8091 Zurich, Switzerland; b Department of ENT, Head and Neck Surgery, Inselspital, Bern University Hospital, University of Bern, Freiburgstrasse 18, CH-3010 Bern, Switzerland; c Head and Neck Anticancer Center, Inselspital, Bern University Hospital, University of Bern, Freiburgstrasse 18, CH-3010 Bern, Switzerland; d Department of ENT, Head and Neck Surgery, Réseau Hospitalier Neuchâtelois (RHNe), Maladière 45, CH-2000 Neuchâtel, Switzerland; e Department of Radiation Oncology, Centre Léon Bérard, 28 Rue Laennec, 69008 Lyon, France; f Department of Radiation Oncology, Inselspital, Bern University Hospital, University of Bern, Freiburgstrasse 18, CH-3010 Bern, Switzerland; g Institute of Pathology, Klinikum Stuttgart, Kriegsbergstr. 60c, 70174 Stuttgart, Germany; h Institute of Tissue Medicine and Pathology, University of Bern, Murtenstrasse 31, 3008 Bern, Switzerland; i Department of Head and Neck surgery, Centre Léon Bérard, 28 Rue Laennec, 69008 Lyon, France

**Keywords:** Head and neck squamous cell carcinoma, Patterns of progression, Lymph node involvement, Neck dissection, Graphical user interface

## Abstract

**Dataset:**

We provide a dataset on lymph node metastases in 968 patients with newly diagnosed head and neck squamous cell carcinoma (HNSCC). All patients received neck dissection and we report the number of metastatic versus investigated lymph nodes per lymph node level (LNL) for every individual patient. Additionally, clinicopathological factors including T-category, primary tumor subsite (ICD-O-3 code), age, and sex are reported for all patients. The data is provided as three datasets: Dataset 1 contains 373 HNSCC patients treated at Centre Léon Bérard (CLB), France, with primary tumor location in the oral cavity, oropharynx, hypopharynx, and larynx. Dataset 2 contains 332 HNSCC patients treated at the Inselspital, Bern University Hospital (ISB), Switzerland with primary tumor location in the oral cavity, oropharynx, hypopharynx, and larynx. For these patients, additional information is provided including lateralization of the primary tumor, size and location of the largest metastases, and clinical involvement based on computed tomography (CT), magnetic resonance imaging (MRI), and/or 18FDG-positron emission tomography (PET/CT) imaging. Dataset 3 consists of 263 oropharyngeal SCC patients underlying a previous publication by Bauwens et al. [1], which were treated at CLB. For these patients, additional information including HPV status, lateralization of the primary tumor and clinically diagnosed lymph node involvement is provided.

**Reuse Potential:**

The data may be used to quantify the probability of occult lymph node metastases in each LNL, depending on an individual patient's characteristics of the primary tumor and the location of clinically diagnosed lymph node metastases. As such, the data may contribute to further personalize the elective treatment of the neck for HNSCC patients, i.e. definition of the elective clinical target volume (CTV-N) in radiotherapy (RT) and the extent of neck dissection (ND) in surgery. There exists only one similar publicly available dataset that reports clinical involvement per LNL in 287 oropharyngeal SCC patients [2]. The data presented in this article substantially extends the available data, it additionally includes pathologically assessed involvement per LNL, and it provides data for multiple subsites in the head and neck region.

Specifications Table

**Table:** 

Subject	Oncology
Specific subject area	Pathological assessment of lymph node metastases in head and neck squamous cell carcinoma
Type of data	Tables
How the data were acquired	The patient information underlying the datasets was acquired during routine clinical care of patients with newly diagnosed HNSCC. All patients received neck dissection either at the CLB or ISB between 2003 and 2019 and resected lymph nodes were investigated by a pathologist. Accompanying clinicopathological information about the primary tumor and the patient as well as clinical lymph node involvement is based on routine clinical diagnosis and staging procedures including CT, MRI, and PET/CT.
Data format	Raw
Description of data collection	The data was collected under the supervision of experienced radiation oncologists and head and neck surgeons by retrospectively analyzing pathology, radiology, and surgery reports retrieved from the patient's medical records.
Data source location	*Institution(s):* 2021-clb-oropharynx: Centre Léon Bérard 2023-clb-multisite: Centre Léon Bérard 2023-isb-multisite: Inselspital, Bern University Hospital
	*City/Town/Region:* 2021-clb-oropharynx: Lyon 2023-clb-multisite: Lyon 2023-isb-multisite: Bern *Country:* 2021-clb-oropharynx: France 2023-clb-multisite: France 2023-isb-multisite: Switzerland
Data accessibility	Repository name: Zenodo, GitHub. Data identification number: • 2021-clb-oropharynx: https://zenodo.org/doi/10.5281/zenodo.10204085 • 2023-clb-multisite: https://zenodo.org/doi/10.5281/zenodo.10210361 • 2023-isb-multisite: https://zenodo.org/doi/10.5281/zenodo.10210423 Direct URL to data: https://github.com/rmnldwg/lydata. The data is provided in three distinct tables, which can be found in their respective folders inside the GitHub repository.

## 1 Background

Treatment of HNSCC patients currently includes elective RT or prophylactic dissection of large parts of the soft neck tissue, which is at risk of harboring occult lymph node metastases that are not clinically/radiologically detectable [3]. Current clinical guidelines on elective nodal RT and neck dissection are mostly based on the prevalence of lymph node metastases in a lymph node level for a given primary tumor location. The overarching goal of this research is to better quantify the risk of occult metastases in each lymph node level based on an individual patient's clinically/radiologically diagnosed state of disease. This may lead to further personalization of neck dissection procedures and the definition of the nodal clinical target volume in RT, also considering the location of clinically/radiologically detected lymph node metastases and characteristics of the primary tumor such as T-category and lateralization of the primary tumor. Detailed datasets reporting lymph node involvement together with clinicopathological factors on a patient-individual level are the basis and a necessary requirement for achieving this goal. This publication presents the largest publicly available such dataset. It can be interactively explored and visualized via the previously developed platform https://lyprox.org.

## 2 Value of the Data


 

• The dataset containing 968 patients represents a substantial addition to a previously published dataset of 287 patients with SCC in the oropharynx [2]. It adds patients with primary tumors located in the oral cavity, hypopharynx and larynx.
• Since pathology after neck dissection is the gold standard for investigating whether occult disease was present in a lymph node, the data is of particularly high quality and an essential addition to the previous dataset [2] reporting only clinical involvement. For parts of the data, both clinical and pathological involvement is provided, containing information on sensitivity and specificity of clinical detection of lymph node metastases.
• Researchers and clinicians working in the field of head and neck cancer, who are interested in the lymphatic spread of the disease and how to manage its related risks may benefit from the publications of these data. The data is the basis for further personalization of neck dissection procedures and elective nodal RT.
• Ultimately, HNSCC patients may benefit from further personalized treatments that better balance the risk of treatment side effects versus the risk of tumor recurrence.
• The data may be used to quantify the probability of occult lymph node metastases in each LNL, depending on an individual patient's characteristics of the primary tumor and the location of clinically diagnosed lymph node metastases.
• Similar to the dataset by Ludwig et al. [2], the three cohorts presented in this work may allow researchers to build and further develop predictive statistical models [4,5] that estimate the risk for occult disease of a patient based on their clinical diagnosis.

## 3 Data Description

The data is provided as three separate datasets that were collected at different institutions. Each dataset is contained in its own directory in the GitHub repository and each directory and data file is structured in the same way. However, some of the information included is specific to one dataset and not contained in the others. Due to these differences, we describe each file separately for completeness. Each dataset is also indexed on Zenodo as their own separate dataset.

### 3.1 2023-isb-multisite/

• **data.csv**: The data is provided as a comma separated value (CSV) containing one row for each of the 332 patients. The table has a header spanning three rows that describe the columns. Below we explain each column in the form of a list with three levels. So, for example, list entry 1.1.7 refers to a column with the three-level header "patient | # | nicotine_abuse" and this column reports about the patient's smoking status:
  1. **patient**: This top-level header contains general patient information.
    1. **#**: The second level header for the patient columns is only a placeholder.
      1. **id**: The local study ID.
      2. **institution**: The institution where the patient was treated.
      3. **sex**: The biological sex of the patient.
      4. **age**: The age of the patient at the time of diagnosis.
      5. **diagnose_date**: The date of diagnosis.
      6. **alcohol_abuse**: Whether the patient was abusingly drinking alcohol at the time of diagnosis.
      7. **nicotine_abuse**: Whether the patient was considered a smoker. This is set to False, when the patient had zero pack-years
      8. **hpv_status**: The p16 status of the patient as a surrogate marker for HPV associated tumors.
      9. **neck_dissection**: Whether the patient underwent a neck dissection. In this dataset, all patients underwent a neck dissection.
      10. **tnm_edition**: The edition of the TNM classification used.
      11. **n_stage**: The pN category of the patient (pathologically assessed).
      12. **m_stage**: The M category of the patient.
      13. **extracapsular**: Whether the patient had extracapsular spread in any LNL.
  2. **tumor**: This top-level header contains general tumor information.
    1. **1**: This second-level header enumerates synchronous tumors. No patient in this cohort had synchronous tumors.
      1. **location**: The location of the tumor.
      2. **subsite**: The subsite of the tumor, specified by ICD-O-3 code.
      3. **side**: Whether the tumor occurred on the right or left side of the mid-sagittal plane.
      4. **central**: Whether the tumor was located centrally or not.
      5. **extension**: Whether the tumor extended over the mid-sagittal line.
      6. **volume**: The volume of the tumor in cm^3.
      7. **stage_prefix**: The prefix of the T category.
      8. **t_stage**: The T category of the tumor.
  3. **CT**: This top-level header contains involvement information from the CT scan.
    1. **info**: This second-level header contains general information about the CT scan.
      1. **date**: The date of the CT scan.
    2. **left**: This describes the observed involvement of the left LNLs.
      1. **<LNL>**: These columns describe the clinical involvement of the left 〈LNL〉, as observed in a CT scan. 〈LNL〉 can take on the values I, Ia, Ib, II, IIa, IIb, III, IV, V, Va, Vb in this dataset. In the event sublevels (like IIa and IIb) were resected and sent to the pathologist separately, the super-level (e.g., II) simply contains the combined information. If the 〈LNL〉 was not separated by sublevel, they are left blank and only the super-level informs whether the 〈LNL〉 was clinically involved or not.
    3. **right**: This describes the observed involvement of the right LNLs.
      1. **<LNL>**: This, in turn, describes the clinical involvement of the right 〈LNL〉, as observed in a CT scan. Again, 〈LNL〉 can be I, Ia, Ib, II, IIa, IIb, III, IV, V, Va, or Vb here.
  4. **MRI**: This top-level header contains involvement information from the MRI scan.
    1. **info**: This second-level header contains general information about the MRI scan.
      1. **date**: The date of the MRI scan.
    2. **left**: This describes the observed involvement of the left LNLs.
      1. **<LNL>**: The same as 3.2.1 but reporting clinical involvement for the respective 〈LNL〉 observed on an MR scan.
    3. **right**: This describes the observed involvement of the right LNLs.
      1. **<LNL>**: The same as 4.2.1 but for the 〈LNL〉 in the right side of the neck.
  5. **PET**: This top-level header contains involvement information from the PET scan.
    1. **info**: This second-level header contains general information about the PET scan.
      1. **date**: The date of the PET scan.
    2. **left**: This describes the observed involvement of the left LNLs.
      1. **<LNL>**: The same as 3.2.1 but reporting clinical involvement for the respective 〈LNL〉 observed on a PET scan.
    3. **right**: This describes the observed involvement of the right LNLs.
      1. **<LNL>**: The same as 5.2.1 but for the 〈LNL〉 in the right side of the neck.
  6. **pathology**: This top-level header contains involvement information from the pathology report.
    1. **info**: This second-level header contains general information about the pathology report.
      1. **date**: Date of the neck dissection.
    2. **left**: Microscopic involvement of the left LNLs.
      1. **<LNL>**: This describes whether the left 〈LNL〉 was pathologically involved or not. As in 3.2.1, the reported 〈LNL〉 are I, Ia, Ib, II, IIa, IIb, III, IV, V, Va, and Vb.
    3. **right**: Microscopic involvement of the right LNLs.
      1. **<LNL>**: The same as 6.2.1, but for the 〈LNL〉 in the right side of the neck.
  7. **total_dissected**: This top-level header contains information about the number of lymph nodes dissected in each LNL.
    1. **info**: This second-level header contains general information about the pathology report.
      1. **date**: Date of the neck dissection.
      2. **all_lnls**: The total number of investigated lymph nodes across all LNLs. Because during some neck dissections multiple LNLs were resected and sent to the pathologist together, this entry may report more investigated LNLs than the sum of each LNL entry separately.
    2. **left**: Number of dissected lymph nodes per LNL on the left side.
      1. **<LNL>**: Number of dissected lymph nodes in the left 〈LNL〉.
      2. **Ib_to_III**: Total number of dissected lymph nodes in the left LNLs Ib-III. Note that this is not just the sum of the dissected nodes in the LNLs Ib to III, because some levels were resected en-bloc. Those are included in this column but could not be resolved for the individual LNLs.
    3. **right**: Number of dissected lymph nodes per LNL on the right side.
      1. **<LNL>**: Total number of dissected lymph nodes in the right 〈LNL〉.
      2. **Ib_to_III**: Total number of dissected lymph nodes in the right LNLs Ib-III. Note that this is not just the sum of the dissected nodes in the LNLs Ib to III, because some levels were resected en-bloc. Those are included in this column but could not be resolved for the individual LNLs.
  8. **positive_dissected**: This top-level header contains information about the number of pathologically positive lymph nodes in each LNL.
    1. **info**: This second-level header contains general information about the findings of metastasis by the pathologist.
      1. **date**: Date of the neck dissection.
      2. **all_lnls**: The total number of investigated lymph nodes that were found to harbor metastatic disease across all LNLs. Because during some neck dissections multiple LNLs were resected and sent to the pathologist together, this entry may report more investigated LNLs than the sum of each LNL entry separately.
      3. **largest_node_mm**: Size of the largest lymph node in the neck dissection in mm.
      4. **largest_node_lnl**: LNL where the largest pathological lymph node metastasis was found.
    2. **left**: Number of pathologically positive lymph nodes per LNL on the left side.
      1. **<LNL>**: Number of pathologically positive lymph nodes in the left 〈LNL〉.
      2. **Ib_to_III**: Total number of dissected lymph nodes found to harbor metastases in the left LNLs Ib-III. Note that this is not just the sum of the dissected nodes in the LNLs Ib to III, because some levels were resected en-bloc. Those are included in this column but could not be resolved for the individual LNLs.
    3. **right**: Number of pathologically positive lymph nodes per LNL on the right side.
      1. **<LNL>**: Number of pathologically positive lymph nodes in the right 〈LNL〉.
      2. **Ib_to_III**: Total number of dissected lymph nodes found to harbor metastases in the right LNLs Ib-III. Note that this is not just the sum of the dissected nodes in the LNLs Ib to III, because some levels were resected en-bloc. Those are included in this column but could not be resolved for the individual LNLs.
  9. **enbloc_dissected**: These columns only report the number of lymph nodes that were resected en-bloc. If, e.g., the LNLs II, III, and IV were resected together, then in each of the respective columns, we report the total number of jointly resected lymph nodes and add a symbol – e.g. 'a' – to identify the en-bloc resection group.
    1. **left**: Number of en-bloc resected nodes on the left side per LNL.
      1. **<LNL>**: Number of lymph nodes resected together that included this level.
    2. **right:** En-bloc resected lymph node count for the right side of the neck.
      1. **<LNL>**: Indicates the number of lymph nodes in the group that included this LNL.
  10. **enbloc_positive**: These columns are structured in the same way as under the key enbloc_dissected, but report the number of lymph nodes that were pathologically involved. Again, the number found in a particular column reports the number of metastatic lymph nodes found in the jointly resected group the respective LNL was part of. LNLs that were resected together share an appended symbol (e.g., “8a”).
    1. **left**: Number of en-bloc resected nodes on the left side per LNL that harbored metastasis.
      1. **<LNL>**: Number of lymph nodes resected together and found to be involved that included this level.
    2. **right**: En-bloc resected lymph node metastasis count for the right side of the neck.
      1. **<LNL>**: Indicates the number of lymph positive nodes in the group that included this LNL.

### 3.2 2023-clb-multisite/

• **data.csv**: The data is provided as a CSV-table containing one row for each of the 373 patients. The table has a header with three levels that describe the columns. Below we explain each column in the form of a list with three levels. So, for example, list entry 1.1.7 refers to a column with the three-level header "patient | # | alcohol_abuse" and underneath it we report each patient's history of alcohol abuse.
  1. **patient**: This top-level header contains general patient information.
    1. **#**: The second level header for the patient columns is only a placeholder.
      1. **id**: The patient ID.
      2. **institution**: The institution where the patient was treated.
      3. **sex**: The biological sex of the patient.
      4. **age**: The age of the patient at the time of diagnosis.
      5. **weight**: The weight of the patient at the time of diagnosis.
      6. **diagnose_date**: The date of surgery because the raw file does not specify a date of diagnosis.
      7. **alcohol_abuse**: Whether the patient was abusingly drinking alcohol at the time of diagnosis.
      8. **nicotine_abuse**: Whether the patient was smoking nicotine at the time of diagnosis.
      9. **hpv_status**: The p16 status of the patient as a surrogate marker for HPV associated tumors.
      10. **neck_dissection**: Whether the patient underwent a neck dissection. In this dataset, all patients underwent a neck dissection.
      11. **tnm_edition**: The edition of the TNM classification used.
      12. **n_stage**: The pN category of the patient (pathologically assessed).
      13. **m_stage**: The M category of the patient.
      14. **extracapsular**: Whether the patient had extracapsular spread. In this dataset, this information is only globally available, not for each individual lymph node level.
  2. **tumor**: This top-level header contains general tumor information.
    1. The second level header enumerates synchronous tumors.
      1. **location**: The location of the tumor. This is empty for all patients, because we can later infer it from the subsite's ICD-O-3 code.
      2. **subsite**: The subsite of the tumor, specified by ICD-O-3 code.
      3. **central**: Whether the tumor is located centrally w.r.t. the mid-sagittal plane.
      4. **extension**: Whether the tumor extended over the mid-sagittal line.
      5. **volume**: The volume of the tumor in cm^3.
      6. **stage_prefix**: The prefix of the T category.
      7. **t_stage**: The T category of the tumor.
  3. **pathology**: This top-level header contains information from the pathology that received the LNLs resected during the neck dissection.
    1. **info**: This second-level header contains general information.
      1. **date**: The date of the pathology report (same as surgery).
    2. **ipsi**: This reports the involvement of the ipsilateral LNLs.
      1. **<LNL>**: This column reports whether the ipsilateral 〈LNL〉 was pathologically involved. In this datasaet, the reported 〈LNL〉 can be Ia, Ib, II, III, IV, V, and VII.
    3. **contra**: This reports the involvement of the contralateral LNLs.
      1. **<LNL>**: This column reports the pathologic involvement of the contralateral 〈LNL〉.
  4. **diagnostic_consensus**: This top-level header is used to indicate that each LNL that was not resected during the neck dissection is assumed to be clinically negative based on consensus decision of all available diagnostic modalities. However, we do not have clinical involvement for resected levels. This means, all columns under this top-level header are inferred from the missing entries under the columns described in point 3.
    1. **info**: This second-level header contains general information.
      1. **date**: The date of the diagnostic consensus (same as surgery).
    2. **ipsi**: This reports the diagnostic consensus of the ipsilateral LNLs.
      1. **<LNL>**: Column reporting the diagnostic consensus of the ipsilateral 〈LNL〉.
    3. **contra**: This reports the diagnostic consensus of the contralateral LNLs.
      1. **<LNL>**: Here, we report the diagnostic consensus of the contralateral 〈LNL〉.
  5. **total_dissected**: This top-level header contains information about the total number of dissected and pathologically investigated lymph nodes per LNL.
    1. **info**: This second-level header contains general information.
      1. **date**: The date of the neck dissection.
    2. **ipsi**: This reports the total number of dissected lymph nodes per ipsilateral LNL.
      1. **<LNL>**: This column reports the total number of dissected lymph nodes in ipsilateral 〈LNL〉. Again, 〈LNL〉 is a placeholder for the reported LNLs and takes on the same values as in point 3.2.1.
      2. **Ib_to_III**: This column reports the total number of dissected lymph nodes in ipsilateral LNL Ib to III.
    3. **contra**: This reports the total number of dissected lymph nodes per contralateral LNL.
      1. **<LNL>**: This column reports the total number of dissected lymph nodes in the contralateral 〈LNL〉.
      2. **Ib_to_III**: This column reports the total number of dissected lymph nodes in contralateral LNL Ib to III.
  6. **positive_dissected**: This top-level header contains information about the number of dissected lymph nodes per LNL that were pathologically found to be positive.
    1. **info**: This second-level header contains general information.
      1. **date**: The date of the neck dissection.
    2. **ipsi**: This reports the number of dissected lymph nodes per ipsilateral LNL that were pathologically found to be positive.
      1. **<LNL>**: Here, we report the number of metastatic nodes in ipsilateral the 〈LNL〉.
      2. **Ib_to_III**: This column reports the number of metastatic dissected lymph nodes in ipsilateral LNL Ib to III.
    3. **contra**: This reports the number of dissected lymph nodes per contralateral LNL that were pathologically found to be positive.
      1. **<LNL>**: And this column reports the number of metastatic lymph nodes in the contralateral 〈LNL〉.
      2. **Ib_to_III**: This column reports the number of metastatic dissected lymph nodes in contralateral LNL Ib to III.

### 3.3 2021-clb-oropharynx/

• **data.csv:** The data is provided as a CSV-table containing one row for each of the 263 patients. The table has a header with three levels that describe the columns. Below we explain each column in the form of a list with three levels. So, for example, list entry 1.i.g refers to a column with the three-level header "patient | # | hpv_status" and underneath it the patients’ HPV status is listed.
  1. **patient**: General information about the patient's condition can be found under this top-level header.
    1. **#**: The second level under patient has no meaning and exists solely as a filler.
      1. **id**: Enumeration of the patients.
      2. **institution**: The clinic where the data was extracted.
      3. **sex**: The biological sex of the patient.
      4. **age**: The age of the patient at the time of diagnosis.
      5. **diagnose_date**: Date of diagnosis (format YYYY-mm-dd) defined as the date of first histological confirmation of HNSCC.
      6. **alcohol_abuse**: true for patients who stated that they consume alcohol regularly, false otherwise.
      7. **nicotine_abuse**: true for patients who have been regular smokers (> 10 pack years), false otherwise.
      8. **pack_years**: Number of pack years of smoking history of the patient.
      9. **hpv_status**: true for patients with human papilloma virus associated tumors (as defined by p16 immunohistochemistry).
      10. **neck_dissection**: Indicates whether the patient has received a neck dissection as part of the treatment.
      11. **tnm_edition**: The edition of the TNM classification used to classify the patient.
      12. **n_stage**: The N category of the patient, indicating the degree of spread to regional lymph nodes.
      13. **m_stage**: The M category of the patient, encoding the presence of distant metastases.
  2. **tumor**: Information about tumors is stored under this top-level header.
    1. **1**: The second level enumerates the synchronous tumors. In our database, no patient has had a second tumor, but this structure of the file allows us to include such patients in the future. The third-level headers are the same for each tumor.
      1. **location**: Anatomic location of the tumor. Since this dataset contains only oropharyngeal SCC patients, this is always oropharynx.
      2. **subsite**: The subsite of the tumor, specified by ICD-O-3 code.
      3. **central**: true when the tumor is located centrally on the mid-sagittal plane.
      4. **extension**: true when the tumor extends over the mid-sagittal plane.
      5. **volume**: The volume of the tumor in cm^3.
      6. **stage_prefix**: Prefix modifier of the T-category. Can be “c” or “p". In this dataset, only the clinically assessed T-category is available.
      7. **t_stage**: T-category of the tumor, according to TNM staging.
  3. **diagnostic_consensus**: This top-level header contains the clinical involvement per-level clinical, representing a consensus decision based on the available diagnostic modalities (typically CT or MRI).
    1. **info**: The second level header contains general information on the diagnostic consensus.
      1. **date**: The date of the diagnostic consensus (same as date of diagnosis).
    2. **ipsi**: These columns report the involvement based on the diagnostic consensus for ipsilateral LNLs.
      1. **<LNL>**: The clinical involvement of 〈LNL〉. In this dataset, the reported LNLs are Ia, Ib, II, III, IV, V, VII.
    3. **contra**: These columns report the involvement based on the diagnostic consensus for contralateral LNLs.
      1. **<LNL>**: Same as 3.2.1 but for the contralateral side of the neck.
  4. **pathology**: Columns under this header contain pathologically assessed involvement information for each LNL.
    1. **info**: The second level header contains general information on the pathology.
      1. **date**: The date of the pathology (same as date of diagnosis).
    2. **ipsi**: Here, we report the ipsilateral LNL involvement based on the pathology.
      1. **<LNL>**: The pathologically assessed involvement of the ipsilateral level 〈LNL〉 lymph nodes. As for the diagnostic consensus under point 3., the reported 〈LNL〉 are Ia, Ib, II, III, IV, V, and VII.
    3. **contra**: The contralateral LNL involvement based on the pathology.
      1. **<LNL>**: The pathologically assessed involvement of the contralateral 〈LNL〉.
  5. **total_dissected**: The total number of lymph nodes resected per LNL.
    1. **info**: The second level header contains general information on the pathology.
      1. **date**: The date of the pathology (same as date of diagnosis).
    2. **ipsi**: Number of dissected lymph nodes in ipsilateral LNLs.
      1. **all**: The total number of lymph nodes dissected in all ipsilateral LNLs.
      2. **<LNL>**: The number of dissected lymph nodes in level 〈LNL〉 only. The placeholder 〈LNL〉 takes on the same values as under point 4.2.1.
    3. **contra**: Number of dissected lymph nodes in contralateral LNLs.
      1. **all**: This column contains the total number of lymph nodes dissected in all contralateral LNLs.
      2. **<LNL>**: This column reports only the number of dissected lymph nodes in the contralateral 〈LNL〉.
  6. **positive_dissected**: The number of metastatic lymph nodes found in the dissected LNLs.
    1. **info**: The second level header contains general information on the pathology.
      1. **date**: The date of the pathology (same as date of diagnosis).
    2. **ipsi**: Columns under this second-level header report the number of metastatic lymph nodes found in the dissected ipsilateral LNLs.
      1. **all**: The total number of metastatic lymph nodes found in all ipsilateral LNLs.
      2. **<LNL>**: The number of metastatic lymph nodes found in the ipsilateral 〈LNL〉 only.
    3. **contra**: Columns under this second-level header report the number of metastatic lymph nodes found in the dissected contralateral LNLs.
      1. **all**: In analogy to the ipsilateral LNLs, this column states the total number of metastatic lymph nodes found in all contralateral LNLs.
      2. **<LNL>**: And these columns reports the number of metastatic lymph nodes found in the contralateral 〈LNL〉 only.

### 3.4 Supporting files

The following supporting files that can be found in each of the three directories:

• <dir>/CITATION.cff: This file specifies how to attribute this dataset and the people who created it. How this file format is defined and how to create e.g., a BibTex citation from it is explained at https://citation-file-format.github.io/.
• <dir>/README.md: A markdown formatted text file that repeats large parts of the data description provided in this article, such that the folder is self-contained. When navigating to the data repository on GitHub https://github.com/rmnldwg/lydata and selecting respective folder 〈dir〉, this README.md is rendered prominently below a list of the files contained in it.
• <dir>/figures/age_and_sex.png: A figure showing the age distribution in the cohort, stratified by sex and smoking status.
• <dir>/figures/t_category.png: Pie chart showing the distribution of patients over T-categories.
• <dir>/figures/subsite.png: Bar plot displaying the number of patients with primary tumors in the different subsites.
• <dir>/figures/bar_plot.png: Stacked bar plot showing the percentage of ipsilateral LNL III involvement as a function of different scenarios and T-category.

### 3.5 Data exploration in LyProx

The datasets can be viewed individually or pooled using the previously developed web-based platform and graphical user interface https://lyprox.org, which has been extended to host the new datasets. The dashboard allows users to select the datasets of interest and filter the patients based on tumor characteristics and lymph node involvement. For example, the user may select all oropharyngeal tumors located in the base of tongue not crossing the midline with involvement of ipsilateral level II. The GUI will then display the number or percentages of patients with involvement in the other levels.

### 3.6 Data characterization

**Fig. 1:**
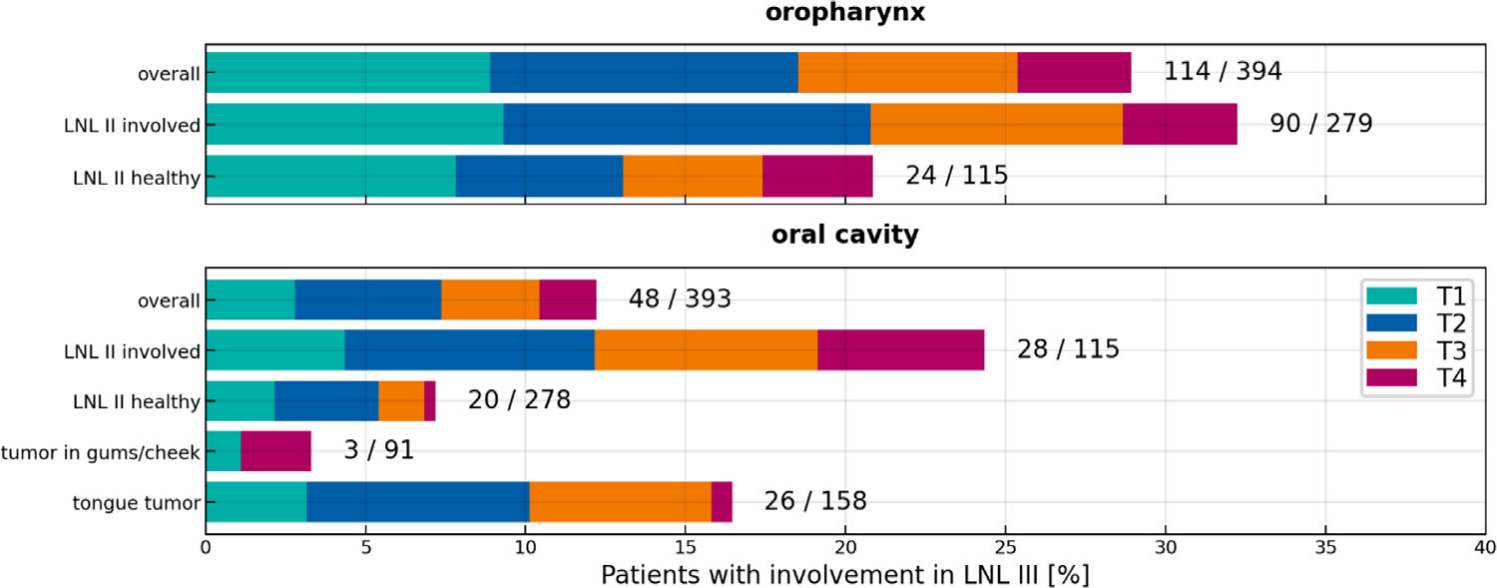
Stacked bar plot reporting the percentage of patients with ipsilateral LNL III involvement found in the three datasets, for oropharyngeal SCC (top panel) and oral cavity SCC (bottom panel). Patients are then stratified by whether LNL II was involved, and by the tumor subsite in the oral cavity case. For each subgroup of patients, the distribution over T-category is shown color-coded. For this plot, all three datasets have been combined. Involvement refers to pathological involvement for most patients (and clinical involvement if levels II or III were part of en bloc resections, such that pathologically positive lymph nodes could not be uniquely assigned to a level).

Fig. 1 illustrates what type of data is provided through this publication and its value for personalizing the risk of lymph node involvement. It shows the percentage of patients with involvement in ipsilateral LNL III for oropharyngeal and oral cavity SCC patients. Previous publications have reported the prevalence of LNL involvement for these tumor locations [6] corresponding to just the first bar in the two panels of Fig. 1. The detailed per-patient and per-level reporting in our datasets allows for stratifying patients according to different risk factors that impact the probability of level III involvement. For example, stratifying oropharynx patients based on involvement of ipsilateral level II shows that relatively few patients have metastases in level III if level II is healthy (24 out of 115 patients, 21%). For patients with metastases in level II, involvement of level III is much more common (90 out of 279 patients, 32%). For oral cavity tumors, the difference in level III involvement depending on presence of metastases in level II is even more pronounced. Similarly, patients can be stratified based on primary tumor subsite, which is illustrated for oral cavity SCC. For tumors located in the gums or cheeks only 3 out of 91 patients have metastases in level III. Instead, for tumors located in the tongue, 26 out of 158 patients have metastases in level III.

**Fig. 2:**
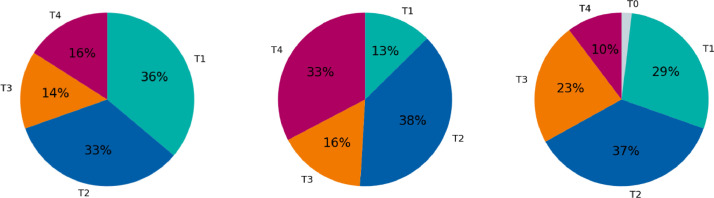
Distributions over T-category in the three datasets, visualized as pie charts. The displayed datasets are – from left to right – the 2023-ISB-multisite data, the 2023-CLB-multisite dataset, and the 2021-CLB-oropharynx cohort.

Fig. 2 characterizes the three patient cohorts in terms of the distribution over T-category. Comparing the ISB and CLB Multisite datasets in terms of T-category shows that CLB dataset is shifted towards more advanced T-category. Compared to the ISB dataset, the CLB data contains fewer T1 tumors and more T4 tumors.

**Fig. 3:**
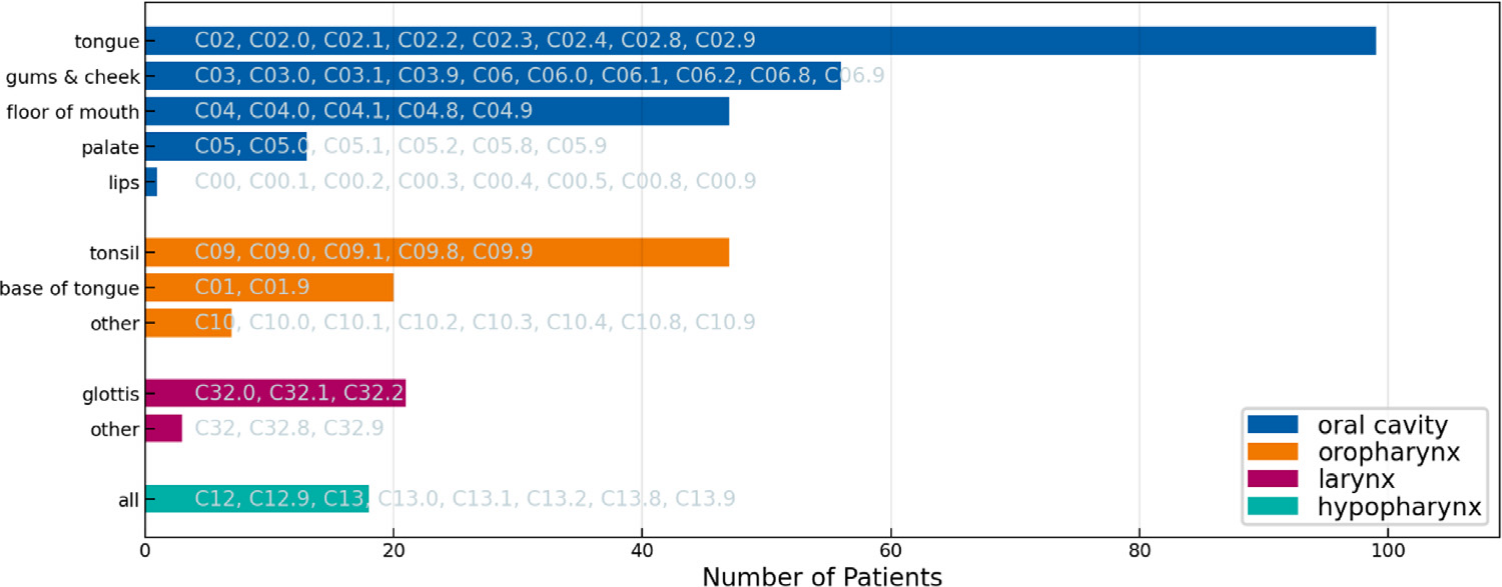
Distribution over primary tumor subsite in the 2023 ISB multisite dataset. For each location, the ICD-O-3 codes that are grouped together are indicated in the figure. E.g., “base of tongue” refers to all patients with an ICD-O-3 codes “C01” or “C01.9”.

**Fig. 4:**
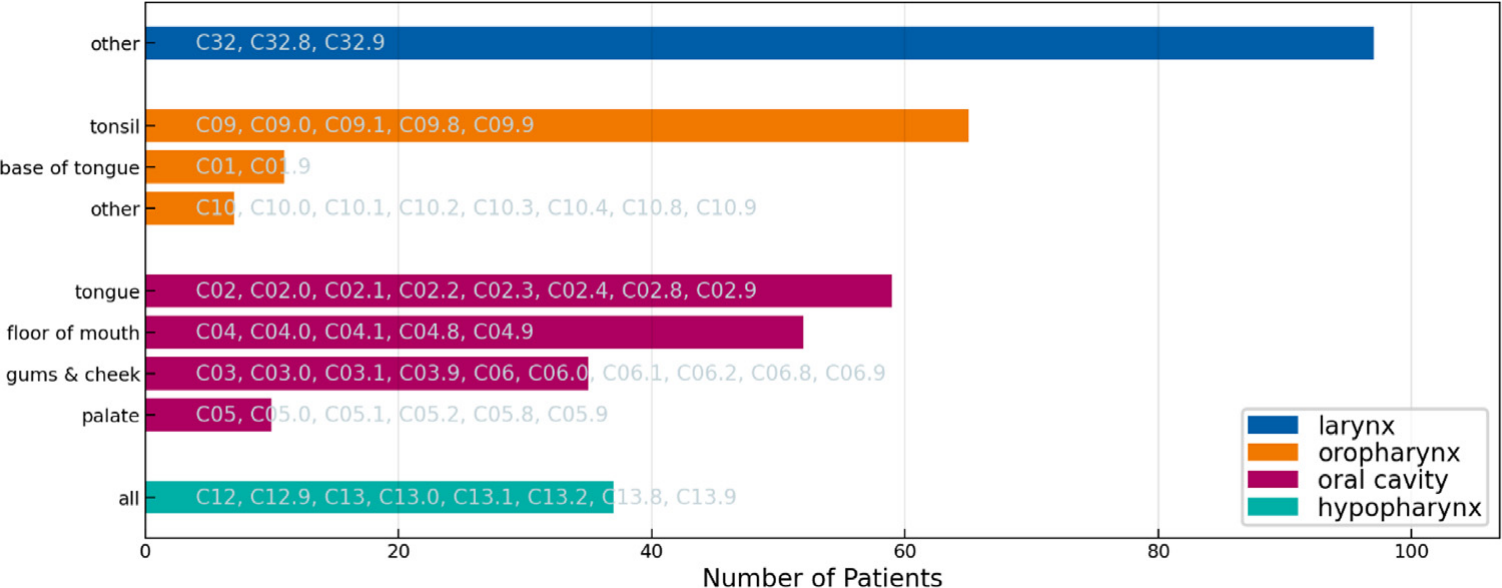
Distribution over primary tumor subsite in the 2023 CLB multisite dataset. For each location, the ICD-O-3 codes that are grouped together are indicated in the figure. E.g., “base of tongue” refers to all patients with an ICD-O-3 codes “C01” or “C01.9”.

**Fig. 5:**
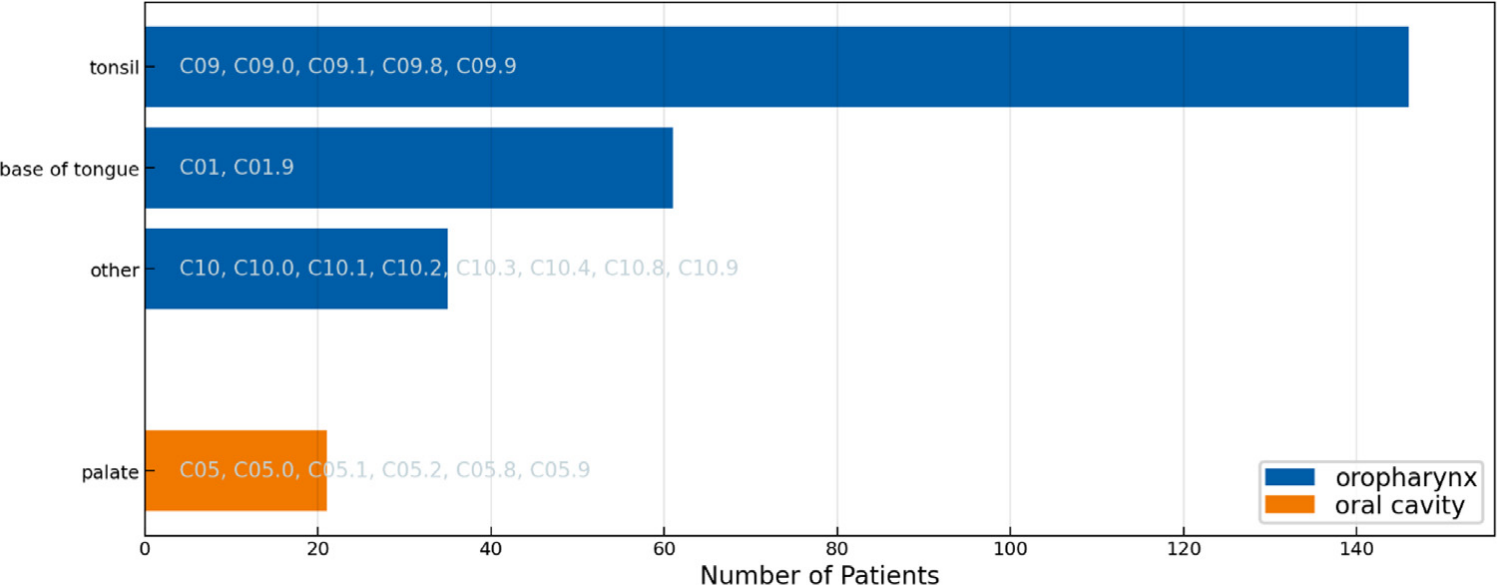
Distribution over primary tumor subsite in the 2021 CLB multisite dataset. For each location, the ICD-O-3 codes that are grouped together are indicated in the figure. E.g., “base of tongue” refers to all patients with an ICD-O-3 codes “C01” or “C01.9”.

Figs. 3, 4, and 5 display the distribution over primary tumor subsites in the three cohorts. The datasets show a similar but not the same distribution across oral cavity and oropharynx subsites. Among oropharynx SCC, tumors located in the tonsil are the most common in all three datasets. Among oral cavity subsites, tumors in the tongue are the most common in both the ISB and CLB Multisite datasets, however, some differences in the distribution are observed. Most notably, the CLB dataset contains a much larger number of larynx SCC. Thus, the figures suggest some differences in patient referral and selecting patients for surgical treatment between the two centers.

## 4 Experimental Design, Materials, and Methods

### 4.1 2023 ISB multisite dataset

#### 4.1.1 Patient cohort

The dataset contains 332 patients with newly diagnosed head and neck SCC who received a neck dissection at the ENT department at ISB between 2010 and 2019. Patients treated with definitive (chemo)radiotherapy to the neck are thus not included in this dataset. Other exclusion criteria are prior treatment to the neck, prior malignancy above the diaphragm, localizations other than those defined of the primary tumor, and skin tumors. The treatment modality was determined individually for each patient, taking into account all available information during the interdisciplinary tumor board. In this context, the extent (side, levels to be removed) of the neck dissection was also determined.

#### 4.1.2 Pathological lymph node involvement

For most patients, dissected lymph node levels were sent to pathology separately. In that case, the pathologist reported the number of investigated and the number of positive lymph nodes per lymph node level. In some cases, neighboring LNL were resected en bloc and not marked individually such that positive lymph nodes could not be uniquely assigned to one LNL. This decision was made by the surgeon during surgery based on the clinical presentation. In many such cases, there was a multilevel involvement of a large metastasis or a conglomerate. In this case, the total number of investigated and positive lymph nodes in the jointly resected levels are reported. Usually, the pathologist also reported the largest lymph node affected by a metastasis and the status of the extracapsular extension (ECE).

#### 4.1.3 Clinical lymph node involvement

Clinical involvement information is based on diagnostic imaging (CT, MRI, PET/CT) acquired during routine clinical care using standard criteria for considering a lymph node as metastatic as described in Biau et al. [3]. The analysis was performed by a specialized head and neck radiologist as part of the interdisciplinary tumor board and thus included in the dataset.

#### 4.1.4 Patient and primary tumor characteristics

The clinical data and information reported in the dataset were taken from the medical records, especially from the tumor board report, which is a consolidation of the available information (medical history, clinical examination, panendoscopy report, results of radiological and pathological examinations). The tumor location, the tumor stage according to the TNM system (7th edition), [7] the relation of the tumor to the midline, HPV status, smoking and alcohol consumption are reported.

### 4.2 2023 CLB multisite dataset

#### 4.2.1 Patient cohort

The dataset contains 373 patients with newly diagnosed head and neck SCC who received a neck dissection at the CLB between 2003 and 2018. Patients treated with definitive (chemo)radiotherapy are thus not included in this dataset. Other exclusion criteria include recurrent tumors or prior treatment of the neck possibly affecting lymphatic drainage. Oropharyngeal SCC patients included in the 2021 CLB oropharynx dataset are not included here, i.e., no patient is duplicated. The extent of neck dissection varied between patients. In almost all patients, ipsilateral levels II, III, and IV were resected; ipsilateral level I was resected in two thirds of patients. Contralateral levels II, III, and IV were resected in approximately half the patients.

#### 4.2.2 Pathological lymph node involvement

Dissected lymph node levels were sent to pathology separated by level. The number of investigated and the number of positive lymph nodes per lymph node level was extracted from the pathology report. In addition, it was recorded whether or not ECE was present anywhere in the surgical specimens, but it was not recorded in which levels the ECE was located.

#### 4.2.3 Clinical lymph node involvement

Clinical involvement was not recorded for this dataset. Based on patterns of care, it is assumed that all lymph node levels that were not resected were clinically node negative.

#### 4.2.4 Patient and primary tumor characteristics

The clinical patient information and tumor location and staging were taken from the medical records. The tumor location was reported as ICD-O-3 codes; the tumor stage according to the TNM system was based on the 7th edition [7] or 8th edition. In addition, smoking history and alcohol consumption are reported, as well as HPV status if available.

### 4.3 2021 CLB oropharynx dataset

The dataset contains 263 patients with newly diagnosed oropharyngeal SCC. All patients received neck dissection at the CLB and two collaborating hospitals. The dataset is underlying the a previous publication by Bauwens et al. [1] investigating differences in lymph node involvement between HPV associated oropharyngeal SCC and HPV negative tumors. Details on how the data was collected are described in the original research article.

## Limitations

While all three provided tables and the rows representing individual patients conform to the same format, they do not all contain the same amount of detail. E.g., in some patients clinical involvement was not separately reported per imaging modality (MRI, CT, …) but as a consensus decision. As another example, not all patients were staged according to the same TNM edition (the used edition is always stated). Also, there are missing values for some patients and some columns. For example, sometimes several LNLs were resected en-bloc during neck dissection, and it was thus not possible to infer which LNL surely must have harbored metastases. The lateralization of the primary tumor, too, is not reported for parts of the cohort. The binary encoding of the reported data itself represents a limitation, as it discards information on the amount of disease and its precise location within an LNL. And lastly, the data may not be representative of the typical distribution of HNSCC patients, both in terms of their primary tumor location and their disease advancement. This is because all patients in these three cohorts have been treated with some form of neck dissection, which is not necessarily performed in all HNSCC patients.

## Ethics Statements

Informed consent was given by all patients included in the dataset. The retrospective data collection at ISB was approved by the cantonal ethics committee of Bern, Switzerland (ID 2018–01,716).

## CRediT authorship contribution statement

**Roman Ludwig:** Software, Data curation, Writing – original draft, Visualization. **Adrian Schubert:** Investigation, Supervision, Writing – review & editing. **Dorothea Barbatei:** Investigation. **Laurence Bauwens:** Investigation. **Sandrine Werlen:** Investigation. **Olgun Elicin:** Writing – review & editing. **Matthias Dettmer:** Resources. **Philippe Zrounba:** Resources. **Panagiotis Balermpas:** Resources, Supervision. **Bertrand Pouymayou:** Data curation. **Vincent Grégoire:** Resources, Investigation, Supervision, Writing – review & editing. **Roland Giger:** Resources, Project administration, Writing – review & editing. **Jan Unkelbach:** Conceptualization, Project administration, Funding acquisition, Supervision, Validation, Writing – review & editing.

## Data Availability

lyDATA: 2023 ISB Multisite (Original data) (Zenodo) lyDATA: 2023 CLB Multisite (Original data) (Zenodo) lyDATA: 2021 CLB Oropharynx (Original data) (Zenodo)
